# An alternative procedure to obtain the mortality rate with non-linear functions: Application to the case of the Spanish population

**DOI:** 10.1371/journal.pone.0223789

**Published:** 2019-10-15

**Authors:** Marcos Postigo-Boix, Ramón Agüero, José L. Melús-Moreno

**Affiliations:** 1 Department of Network Engineering, Universitat Politècnica de Catalunya, Barcelona, Spain; 2 Communications Engineering Department, University of Cantabria, Santander, Spain; UCIBIO-REQUIMTE, Faculty of Pharmacy, University of Porto, PORTUGAL

## Abstract

This paper presents an alternative calculation procedure to calculate the mortality rate, exploiting the data available in the Eurostat demography database for Spain. This methodology has been devised based on two of the most widely known and widespread models to establish the mortality rate: The Gompertz-Makeham (GM) and Lee-Carter (LC) models. Our main goal is to obtain a model yielding a similar accuracy than LC or GM, but able to capture the variation of their parameters over time and ages. The method proposed herewith works by applying simple or double fitting, with non-linear functions, to the values of the parameters considered by each one of such models. One of the main advantages of our approach is that we considerably reduce the amount of data that is required to establish the mortality rate, with respect to what would be needed if the traditional models were used. On the other hand, it also allows analyzing the evolution of the mortality rate, even if no real data was available for a particular year. The results evince that, besides fulfilling the two aforementioned goals, the proposed scheme yields an estimation error that is comparable with that offered by the traditional approach.

## Introduction

The study of social networks is one of the most active research fields at the time of writing. Researchers from different disciplines such as, sociology, demography, psychology, engineering, computing, etc., are paying attention to them. The large number of contributions that are regularly presented in this field are focused on analyzing, carrying out and studying all types of multidisciplinary cases over different scenarios, where these social networks could be developed and deployed. In this sense, the impact that these networks have on the life and daily activities of millions of people is remarkable. In this context, this proposal arises from the authors’ attention towards the study and analysis of familiar social networks. One of the first aspects to tackle is the generation of family structures to afterwards analyze the ways to establish the relationships among the members of the family structures.

The work discussed in this paper is included in a first phase, which involves the generation of family structures in an ad-hoc simulation platform, and it is actually focused on one of the needed modules to simulate these structures. In such process it becomes essential to have a procedure that allows to know how these individuals are dying, considering their gender. This might have a clear impact on the development of the family structures. We introduce a module that tackles this task, reproducing the mortality rate of these individuals and allowing to establish an estimation of its future evolution. Our requirements in the development of this module were to deploy an alternative procedure to generate the mortality rate with less data than that required by the underlying models and so reducing the complexity of the simulation when computing deaths in family members. This goal requires using simple tools, yet offering an accurate behavior. The obtained results show that the absolute mean error observed for the proposed method is alike the ones seen for the underlying models, and acceptable with respect to the real data. For illustrative purposes, we use statistical data from the Eurostat (the statistical office of the European Union that provides high quality statistics for Europe) demography database [1] for Spain. However, our proposal can be straightforwardly applied to any other data.

The advantage this proposal introduces is that the calculation requires less data, and it is more scalable, due to the use of non-linear fitting functions. Furthermore, it is possible to estimate the future evolution of the mortality rate, without requiring the real values. Amongst the various methods that are proposed in the literature to evaluate the mortality rate, two of the most widespread ones, Gompertz-Makehan (GM) [2,3] and Lee-Carter (LC) [4] models, were selected as the underlying ones to build our methodology. This paper does not consider extensions to such models, as those that have been proposed to address some of their original limitations, such as [5–13] for the LC model, or [14–17] for the GM model. Rather, our main interest has been to propose a procedure that facilitates the calculation of the mortality rate, reducing the amount of information to be used and yielding more versatility in the whole process.

The results, compared with the real mortality rates of the Spanish population during the last 35 years from the Eurostat demography database [1], confirm that our proposed procedure yields similar values to the aforementioned models. It first calculates the parameters that are required by each model, and then they are fitted at every age and for every year for both LC and GM, by means of simple or double fitting with polynomial functions over their ranges, as will be explained later in Section 3. These non-linear functions were selected since they offer better fitting as the degree of the polynomial increases. They are well known, easy to work with, and they have some scalability properties with a linear cost, specified as the number of needed data. In our case, as it has been previously said, our aim is not to improve the accuracy of such underlying models, but just confirming that our proposal yields a similar performance. Although working with precise mortality rates is always interesting, we claim that it is not essential for generating families with a simulator, since we would rather foster a procedure that is easy to use and features scalability and versatility, to reduce the complexity of the simulations. As will be seen later, the mortality rates that can be obtained with the proposed method are rather similar to those obtained by the original models, and even when compared with the real mortality rates from the Eurostat demography database [1].

It is also important to remark that this proposal does not aim at replacing the original models, but rather at using them to offer an alternative mortality rate calculation, which yields a reduction in the amount of used data. It rather captures the evolution of the parameters of the LC or GM models, against time or ages. In addition, it also brings more flexibility and scalability, since it allows to choose the degree of complexity of the fitting curves, with a trade-off between information requirements and accuracy. That is, if the degree of the fitting function was high, the approximation obtained would be better, but the corresponding cost, regarding the amount of required data, would linearly increase.

In particular, the objectives pursued with this proposal are: (1) description of the alternative procedure for both genders, taking as an initial reference the models proposed by GM and LC., and (2) presentation of the obtained results, which includes an evaluation of the corresponding errors with respect to the real data. The benefits of our proposal are a notable reduction of the amount of required data, and its large scalability, due to the use of fitting parameters with nonlinear functions and, finally, the possibility to capture the evolution trend of the mortality rate.

In the rest of the paper, first, we discuss some related works, identifying the main differences with the approach we introduce herewith. We describe the proposed procedure for obtaining mortality rates from the selected models of GM and LC, using fitting functions to approximate their corresponding parameters. We discuss the results obtained when applying the proposed methodology, comparing them with those of the underlying approaches that were used. Finally, we conclude the paper and provide an outlook of how we plan to extend this line of research.

## Related work

The research community has paid significant attention to the study of mortality rates. Related works encompass both original models [2–4], as well as other approaches that extend or modify these, to overcome certain limitations or to allow more accuracy when estimating the mortality rate, for example, for certain age range. Some illustrative examples of this last group are [5–17]. The large number of works within this research realm is a consequence of how different business sectors (insurance companies, public services, etc.) value the corresponding results.

The interest of our proposal, focused on an alternative methodology to establish the mortality rate is to strongly facilitate its use. This particular feature substantially differs from the interests of most of existing works [5–14]. Those deal, generally speaking, with the introduction of conceptual variations of the original versions of the models. We proceed in a different way, since we do not aim to modify these original models per se, but rather to introduce a new way of estimating their parameters to reduce the amount of data needed to obtain the mortality rates comparing to GM [2,3] and LC [4].

On the other hand, we argue that the proposed methodology is generic enough to be applied with variations of the original models, as those presented in such papers, and it is therefore worth analyzing them. In this initial contribution, we focus on the original models, GM, which is discussed in [2,3], and LC, whose description is given in [4].

Such models have been shown to approximate quite well the mortality rate values, and we therefore understand that they are accurate enough to fulfill our ultimate requirement, which is their use to develop a tool to generate artificial family structures, which would be then used to build social network models. In this scenario we do not require a high level of precision in the mortality rate values, as it would be the case of other applications (social insurances, public sector), where the goal should clearly be on the accuracy of the models. This is because the models used in the simulator should yield enough accuracy, but more lightweight, in terms of the amount of required data.

Hence, we do not seek an improvement of the precision of the original models, that we deem precise enough for our particular needs, but rather to maintain their accuracy, while reducing the amount of information that is required to use them. Another advantage would be the possibility of exploiting the proposed method as a means to predict mortality rates without real data, since it actually captures the evolution through the years.

Other approaches worth mentioning are those from Wilmoth et al. [18] and Clark [19]. They also seek reducing the complexity of more traditional models, yet yielding accurate mortality rates. The authors of [18] propose using child and adult mortality to estimate life tables. On the other hand, Clark's model is based on Singular Value Decomposition (SVD). While this indeed reduces the number of parameters, it also requires some pre-processing of the data (age grouping, data smoothing). In any case, none of them aims at capturing the relationship with the year, nor the mortality rate trend, which is one of the main goals of the approach proposed herewith.

On the other hand, there exist various works worth mentioning, since they illustrate how this line of research has evolved during the latest years. In this sense, the authors of [20,21] provide a survey of the most interesting mortality rate models, and the modifications that have been proposed since 1980. They review several proposals, which are then classified according to three broad categories: (1) models based on expectations, often managed by experts’ opinions; (2) those that use extrapolation techniques, which are probably the most widespread ones; and finally, (3) those that suggest to tackle the analysis of mortality rate from the perspective of risk situations. Other papers that also analyze modeling and prediction techniques for mortality rate are [22–27] and references therein. Other texts that may be of interest are [28,29] and references therein.

## Proposed approach

Traditional mortality rate models are based on various parameters, as we previously discussed. In particular, both Lee-Carter and Gompertz-Makeham equations each use three parameters.

In the case of the LC model, the mortality rate (ω_LC_) can be estimated as:

$$\text{log}(\omega_{LC}\left(x,t\right))=a\left(x\right)+b\left(x\right)k\left(t\right)$$

where x and t correspond to the particular age and year, respectively, and a, b, and k are the three LC model parameters.

For the GM model, we will use the following expression:

$$\omega_{GM}\left(x,t\right)=\alpha \left(t\right)+\beta \left(t\right)e^{\gamma \left(t\right)\;x}$$

where x and t also correspond to the particular age and year, respectively, and α, β, γ, are the model three parameters.

The traditional way of using such models is taking a dataset for a particular year (the real mortality rate of a particular region/country and gender), and employ a fitting procedure to find the combination of parameters that yield the most accurate results. Hence, if we wanted to exploit the model for several years, it would actually require having the corresponding data sets for all of them (thus, needing much more information), and carrying out the corresponding individual fitting. In the case of GM, the three parameters depend on the particular year, while for LC, two of them depend on the year, while the third one is established for all ages.

There are two main disadvantages of this approach: (1) the amount of data that is required to use them; (2) neither of the two models can be straightforwardly used to predict the mortality rates for years in which real data is unknown, since they do not capture any temporary evolution of the corresponding parameters.

The main contribution of the scheme we present herewith is that we leverage a novel approach, by means of which we establish a relationship between the two models’ parameters (three for each of them) and the current year (and age for the LC solution). In this way, we generalize its use, since by having just the year/age as inputs (since for the LC model we need the corresponding age), we could derive the mortality rate. This indeed tackles the two limitations that we previously identified. (1) We do not need to have too much information to use the model, once it has been established. (2) We could even use this approach to ‘predict’ the mortality rate even for years we do not have real data yet, since we are capturing its variation with the years.

Below we describe the steps that we follow to build the proposed methodology: preprocessing statistical data, application of a mortality model for each year, capturing the variation of the mortality models’ parameters along years, and evaluation of the whole approach.

## Preprocessing statistical data

First, we start by selecting the datasets that we will use to train the two models. In our case, we are taking the information provided by Eurostat. We focus on our country (Spain) to illustrate our methodology, which can be anyway applied to any other dataset. We use data spanning from 1981 to 2016 for both male and female populations. We first get from Eurostat two parameters: *D(x*,*t)*, which corresponds to the number of deaths for a given gender in the corresponding square at completed age *x* in year *t*, and *P(x*,*t)*, the cohort on 1 January (year *t*) of the population of a given gender at completed age *x*. The mortality rate at completed age *x* in year *t*, *m(x*,*t)*, is then calculated as follows:

$$m\left(x,t\right)=\frac{D\left(x,t\right)}{\left(P\left(x,t\right)+P\left(x+1,t\right)\right)/2}$$

**Fig 1:**
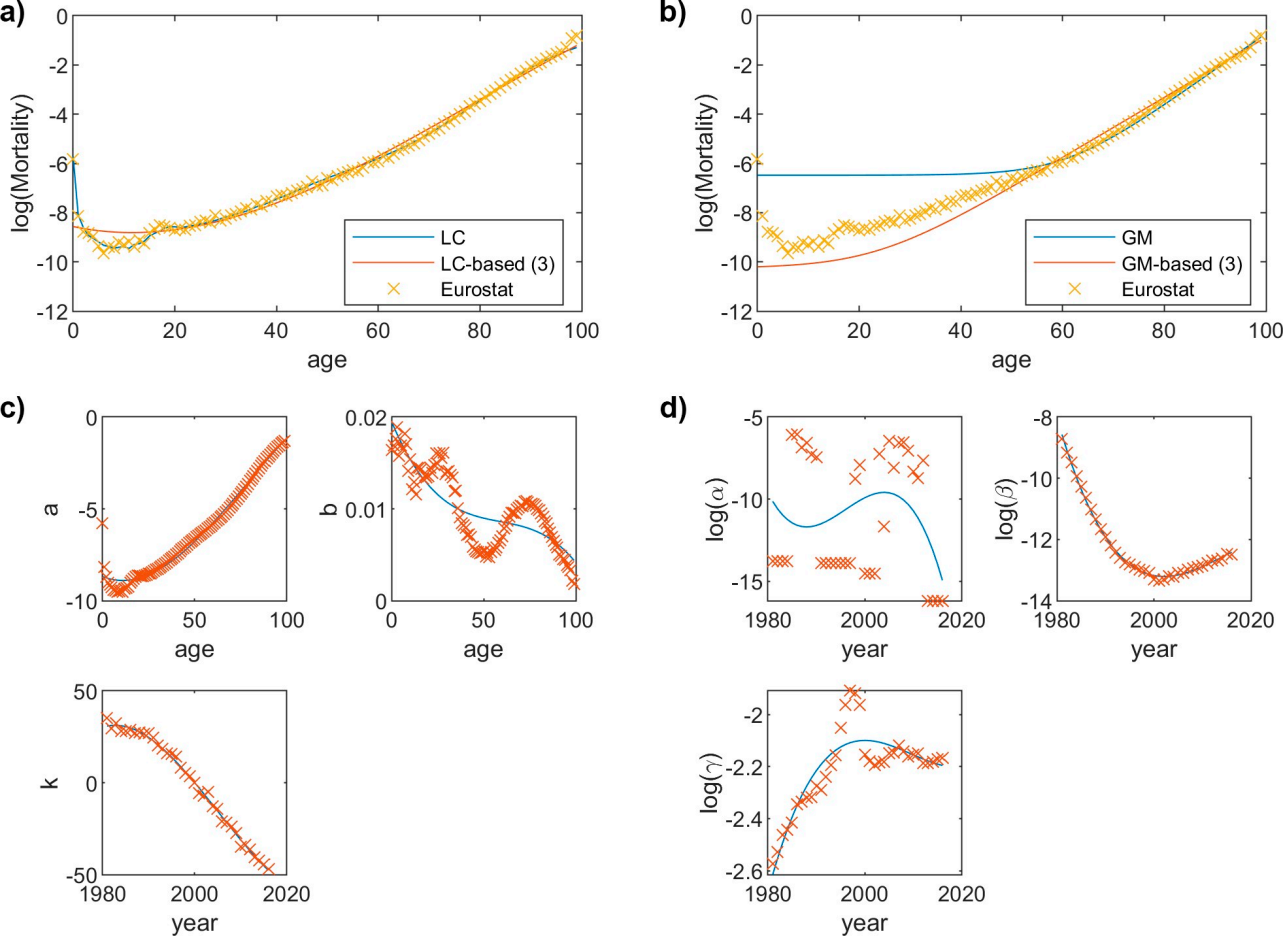
Example of the proposed approximation of the mortality rate. We use real data from Eurostat for female gender in 1998 (Spain). The obtained mortality rates are represented in panels (a) and (b) with cross markers. Panel (a) also shows the approximation with the LC model and our proposed LC-based approximation. Panel (b) also shows the approximation with the GM model and our proposed GM-based approximation. Panel (c) and (d) show how we model the three parameters with the fitting function (third degree polynomial) for the LC-based and GM-based models, respectively.

Fig 1C and 1D show the mortality rates in 1988 for female gender, obtained from Eurostat.

## Application of a mortality model

Using Matlab and its curve-fitting toolbox [30], we fit the LC or GM models to the mortality values from Eurostat. We apply a curve fitting process (minimizing the Least Square error) to establish the values for their corresponding parameters. For the GM case, we obtain α, β, and γ for each year, and for the GM case, a and b for each age and k for each year. Fig 1C and 1D show the LC fitting and GM fitting, respectively, for female mortality rates in 1998.

## Model of the variation of the mortality models parameters

After carrying out such fitting for all the considered years (and ages, for the LC case), we establish how the three parameters (in each model) vary against the year (and age for the case of the a and b parameters in LC). Afterwards, we approximate such variation, again using a curve fitting process.

This is the most critical part of the proposed scheme, since there is not a predefined function to carry out such fitting. Hence, by analyzing the actual values of such parameters, we were able to establish some sensible choices for the fitting functions. In the case of GM case, all three parameters were positive, and we thus decided to use an exponential function to fit them. We generalize the exponent of the fitting functions ($\hat{\alpha },\;\hat{\beta },\;\hat{\gamma }$) with a polynomial of degree *n*:

$$\hat{\alpha }=e^{\alpha_{p_{0}}+\alpha_{p_{1}}t+\ldots +\alpha_{p_{n}}t^{n}}\Rightarrow \text{log}\left(\hat{\alpha }\right)=\alpha_{p_{0}}+\alpha_{p_{1}}t+\ldots +\alpha_{p_{n}}t^{n}$$

$$\hat{\beta }=e^{\beta_{p_{0}}+\beta_{p_{1}}t+\ldots +\beta_{p_{n}}t^{n}}\Rightarrow \text{log}\left(\hat{\beta }\right)=\beta_{p_{0}}+\beta_{p_{1}}t+\ldots +\beta_{p_{n}}t^{n}$$

$$\hat{\gamma }=e^{\gamma_{p_{0}}+\gamma_{p_{1}}t+\ldots +\gamma_{p_{n}}t^{n}}\Rightarrow \text{log}\left(\hat{\gamma }\right)=\gamma_{p_{0}}+\gamma_{p_{1}}t+\ldots +\gamma_{p_{n}}t^{n}$$

As will be discussed later, the results were rather good.

On the other hand, this was not the case for the LC parameters, and we thus decided to try both with exponential and polynomial fitting functions. Higher order polynomials would likely yield more accurate fittings, but would also increase the complexity of the proposed model, requiring a higher number of parameters. We discuss this trade-off in the results section. The polynomial functions to fit the LC parameters are the following:

$$\hat{a}=a_{p_{0}}+a_{p_{1}}t+\ldots +a_{p_{n}}t^{n}$$

$$\hat{b}=b_{p_{0}}+b_{p_{1}}t+\ldots +b_{p_{n}}t^{n}$$

$$\hat{k}=k_{p_{0}}+k_{p_{1}}t+\ldots +k_{p_{n}}t^{n}$$

We repeat the fitting process iteratively. Hence, we start with one parameter, to find the fitting function. Then, we re-start the whole process again, but actually using the previously found fitting to derive such parameter. We tried the 6 different combinations that were possible, and we selected the one that yielded the lowest error. Fig 1C and 1D show the LC-based fitting and GM-based fitting for the mortality rates of females in 1998, respectively. On the other hand, Fig 1A and 1B show the fitting of the models’ parameters.

## Evaluation of the new mortality model

Finally, once we have a fitting function for all the parameters, we calculate the corresponding mortality rate for all the years used as training datasets. Then, we established the corresponding errors, compared with both the real data and the values that the traditional fitting process would have yielded (i.e. by establishing the parameters on a yearly basis for the GM model and using the age for the LC approach).

For illustration purposes, a summary of the whole process for both models (LC and GM, respectively) is shown in Fig 1. We use a third degree polynomial for the fitting process of the models’ parameters. We have used data from 1988 and mortality rates for the female gender. Fig 1A and 1B show the mortality rates obtained from Eurostat Data, the approximation with the legacy LC and GM models, and our proposed LC-based and GM-based approximation. Fig 1C and 1D depict how the selected fitting function (third degree polynomial) approximates the corresponding parameters. As can be seen (and will be discussed in the next section) the accuracy of the LC model is higher, although it is worth noting that we are plotting the logarithm of the mortality rate, and that both models (especially GM) show a worse behavior for capturing the mortality rates of young population. This loss of accuracy for infant mortality is a direct consequence of the underlying models. While this might be relevant in certain applications (insurance, health-related studies), we claim that it might not be such significant for our ultimate purpose, which is social relationships, and their impact on communication habits, not relevant until elder ages. We might have expected this behavior, since the information we have is less statistically precise.

## Results

In order to validate the proposed methodology, we compare in this section the accuracy of the traditional models (LC and GM) with that shown by the polynomial approximations (LC-based and GM-based), by means of the absolute error (AE), with respect to the real values obtained from the Eurostat datasets.

We recall that we can define the absolute error (AE) as follows:

$$AE\left(x,t\right)=\left|\hat{m}\left(x,t\right)-m\left(x,t\right)\right|$$

Where *m(x*,*t)* is the real mortality rate, taken from the Eurostat data, and $\hat{m}\left(x,t\right)$ is the value yielded by the corresponding models. Then, in order to establish the accuracy of the different models, we use the Mean Absolute Error (MAE), which calculates the mean of all the AE values. Hence, MAE stands as an indicator of the overall model accuracy, being easier to understand than Root Mean Square Error (RMSE) [31]. We represent the mortality rate in two dimensions (*t*: years, *x*: ages) and we can thus obtain three different MAE measures: 1) MAE for different ages, 2) MAE for different years, and 3) a global MAE. As mentioned earlier, we are using Spain mortality rates to assess the feasibility of our proposed methodology. The available data ranges from *t*_1_ = 1981 to *t*_36_ = 2016, and entails ages from *x*_1_ = 0 to *x*_100_ = 99. Hence, we can structure the original data as a 100 x 36 matrix.

The next subsection exploits the AE measure as a means to represent the accuracy of the various models. Afterwards, we combine them into the global MAE, and we study it for different ages and years.

## Absolute error

**Fig 2:**
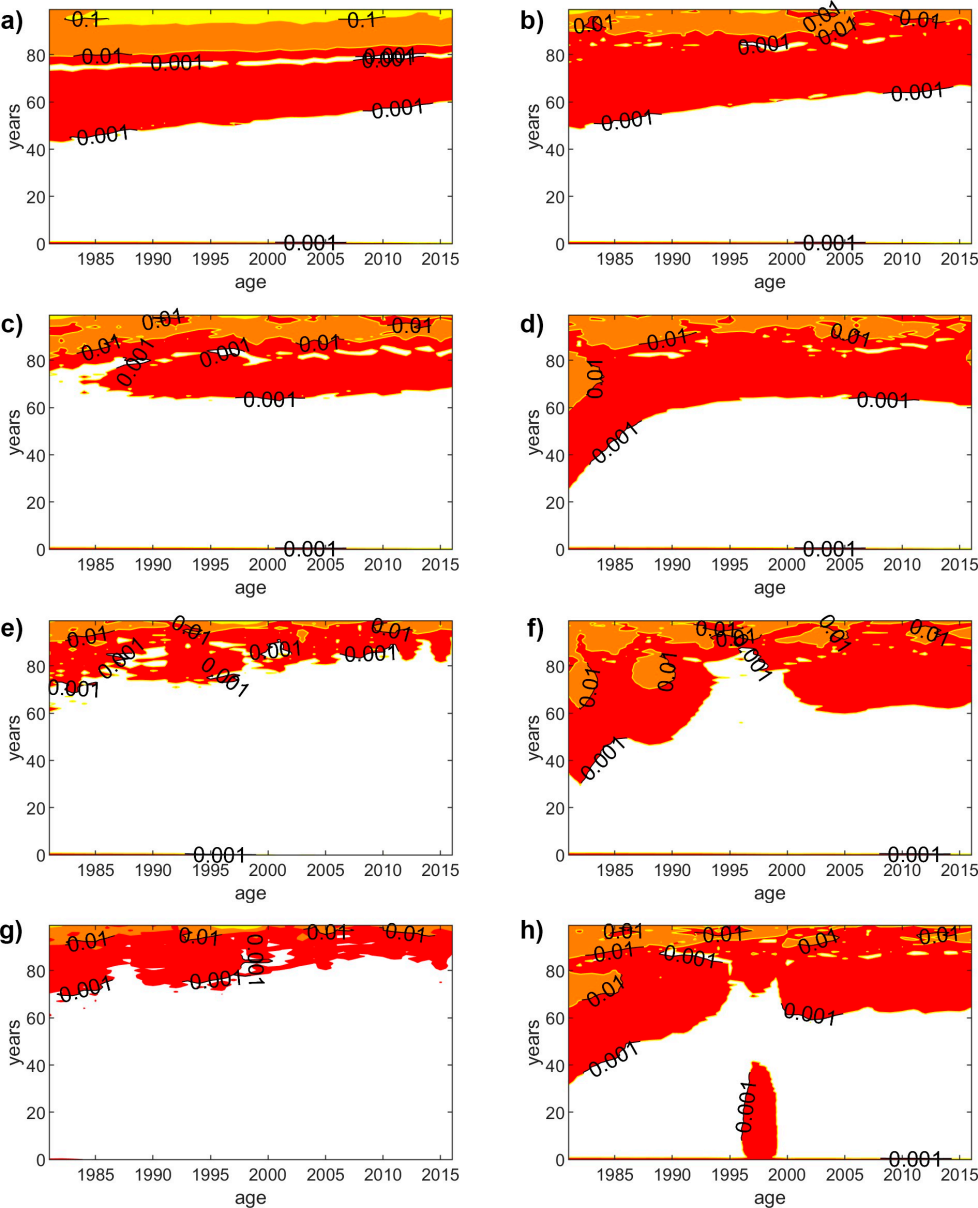
Absolute error (females). Contour plot, which represents the isolines of the AE on the years-age plane for the LC and GM models, as well as for the proposed methodology (using polynomials of order 1, 3 and 9 for the parameter fitting functions) for female population. The isolines show the points with same AE. We show isolines for AE = {0.001, 0.01, 0.1, 1} and the colors represent the values between two isolines ([0.001-RED-0.01-ORANGE-0.1-YELLOW-1). (a) LC-based (polynomial order = 1). (b) GM-based (polynomial order = 1). (c) LC-based (polynomial order = 3). (d) GM-based (polynomial order = 3). (e) LC-based (polynomial order = 9). (f) GM-based (polynomial order = 9). (g) LC model. (h) GM model.

**Fig 3:**
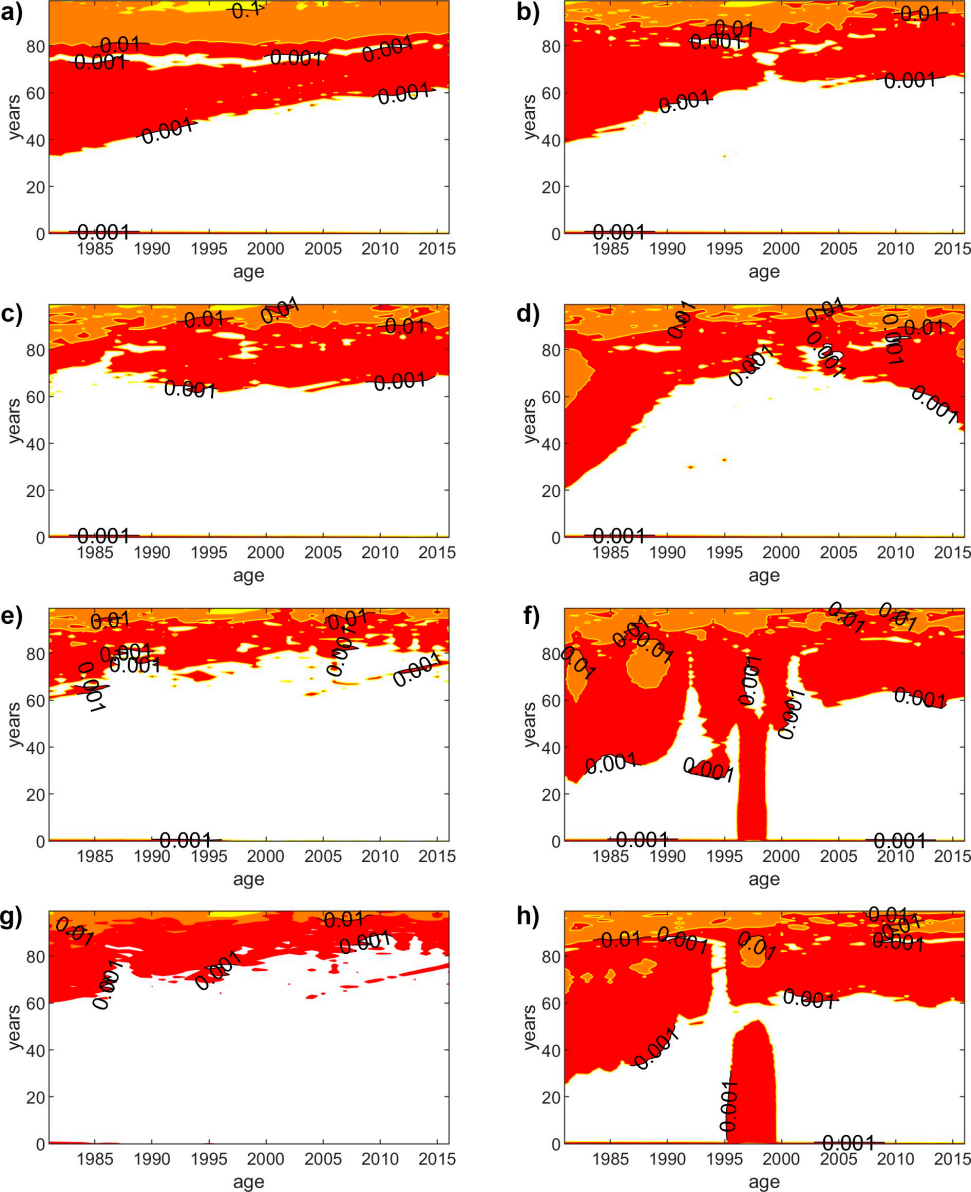
Absolute error (males). Contour plot, which represents the isolines of the AE on the years-age plane for the LC and GM models, as well as for the proposed methodology (using polynomials of order 1, 3 and 9 for the parameter fitting functions) for female population. The isolines show the points with same AE. We show isolines for AE = {0.001, 0.01, 0.1, 1} and the colors represent the values between two isolines ([0.001-RED-0.01-ORANGE-0.1-YELLOW-1). (a) LC-based (polynomial order = 1). (b) GM-based (polynomial order = 1). (c) LC-based (polynomial order = 3). (d) GM-based (polynomial order = 3). (e) LC-based (polynomial order = 9). (f) GM-based (polynomial order = 9). (g) LC model. (h) GM model.

Fig 2 shows the absolute error for the traditional LC and GM models, as well as for the proposed methodology (using polynomials of order 1, 3 and 9 for the parameter fitting functions) for female population. We use a contour plot, which represents the isolines of the AE on the years-age plane. As we could have expected, the absolute error reduces when the degree of the polynomial fitting function increases. On the other hand, the accuracy slightly worsens for higher ages, although it is still rather acceptable. Fig 3 shows the same results for male population, but we do not include the linear fitting function (degree = 1), since the results are alike for the women case. By analyzing the results of all cases, we can extract some interesting conclusions. Generally, we can state that the accuracy of LC is slightly better, but GM yields lower errors for higher ages, where the mortality rate might have a more relevant impact. If we compare the results for both genders, the results yield a slightly higher error for men. There exists a particular situation worth discussing. We can see in Fig 3 that GM exhibits a worse accuracy for young ages within the 1997–1998 year range. Surprisingly, this is not caught when the fitting functions have a lower degree (degree = 3), but the higher precision of the higher order fittings (degree = 9) leads to capture such inaccuracy, which is a consequence of the worse behavior of the original GM model, as can be seen in Fig 3.

## MAE over years dimension

We now analyze the MAE over the years dimension (*t*) for a particular age *x*, which is defined as follows:

$$MAE_{x}=\frac{1}{T}\overset{t_{T}}{\underset{t=t_{1}}{\sum }}\left|{\hat{m}}_{x,t}-m_{x,t}\right|$$

where $\hat{m}\left(x,t\right)$ is the mortality rate obtained when using a model, *m(x*,*t)* is the real mortality rate (as taken from the Eurostat information), and *T* is the number of considered years, which in our case are the 36 years, from which we have real information in Eurostat.

**Fig 4:**
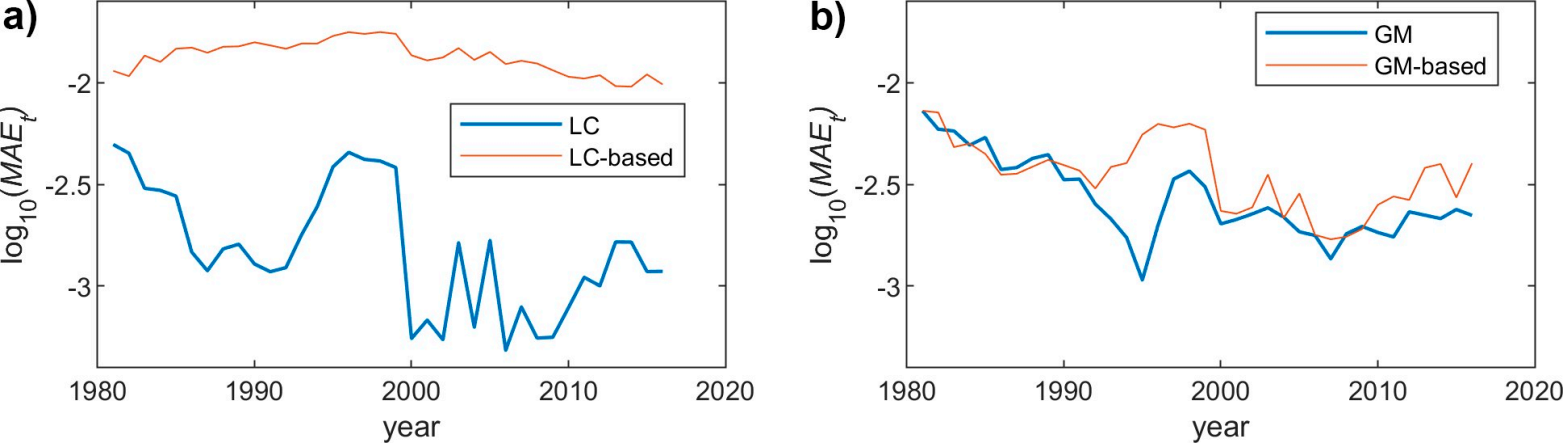
MAE (females) vs. year. Polynomial order = 1. (a) LC and LC-based models. (b) GM and GM-based models.

**Fig 5:**
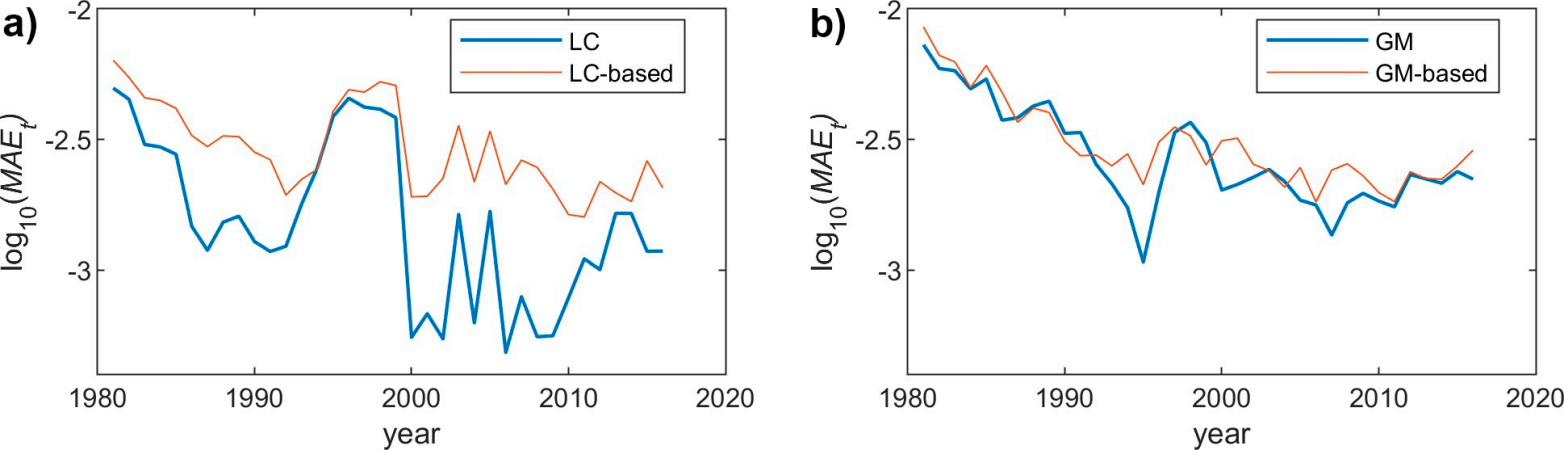
MAE (females) vs. year. Polynomial order = 3. (a) LC and LC-based models. (b) GM and GM-based models.

**Fig 6:**
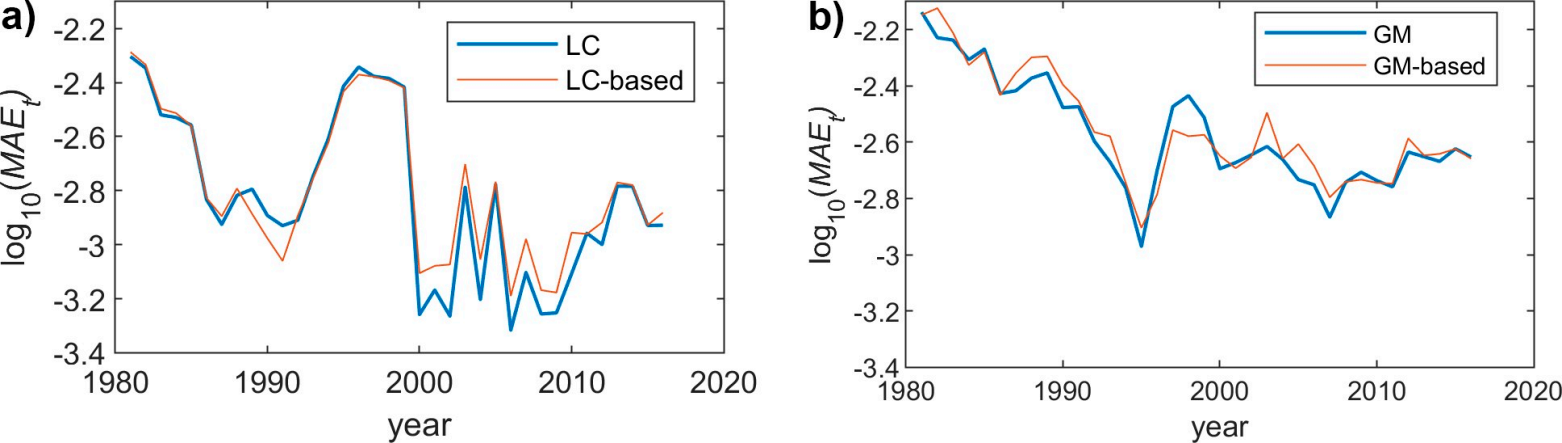
MAE (females) vs. year. Polynomial order = 9. (a) LC and LC-based models. (b) GM and GM-based models.

Since we have already seen that the behavior of the proposed scheme is rather similar for both genders, we will just represent the results obtained for female population. Figs 4, 5 and 6 show the MAE_X_ for the two models (LC and GM) and the different fitting functions (changing the polynomial degree). The most interesting conclusion is that the impact of the model complexity (i.e. degree of the polynomial fitting function) is much more relevant for the LC case. In this sense, the observed MAE_X_ for the linear fitting is rather high in LC, while the accuracy when using a 9-degree function is quite high. On the other hand, in GM, the precision of the approximation, even if it improves when we increase the degree, is even acceptable for the simplest fitting choice (degree = 1).

## MAE over ages dimension

We now change the dimension, and we calculate MAE over the age dimension (*x*) for a particular age *t*, as follows:

$$MAE_{t}=\frac{1}{X}\overset{x=x_{X}}{\underset{x=x_{1}}{\sum }}\left|{\hat{m}}_{x,t}-m_{x,t}\right|$$

where $\hat{m}\left(x,t\right)$ is the mortality rate obtained when using the proposed model, *m(x*,*t)* the real mortality rate (taken from Eurostat data), and *X* is the number of considered ages (100 in our analysis).

**Fig 7:**
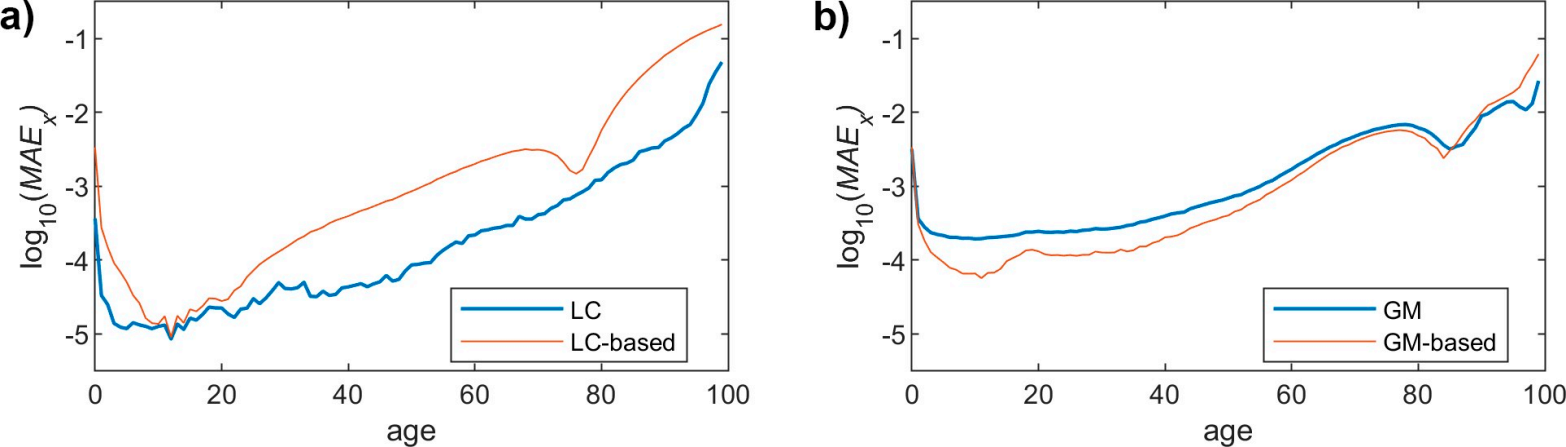
MAE (females) vs. age. Polynomial order = 1. (a) LC and LC-based models. (b) GM and GM-based models.

**Fig 8:**
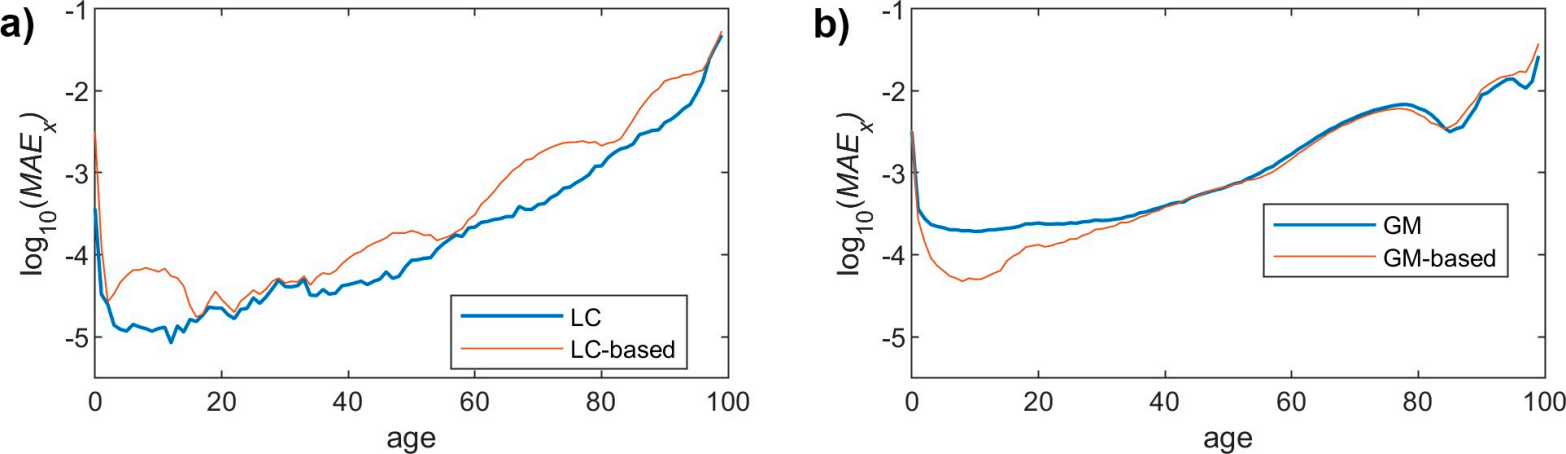
MAE (females) vs. age. Polynomial order = 3. (a) LC and LC-based models. (b) GM and GM-based models.

**Fig 9:**
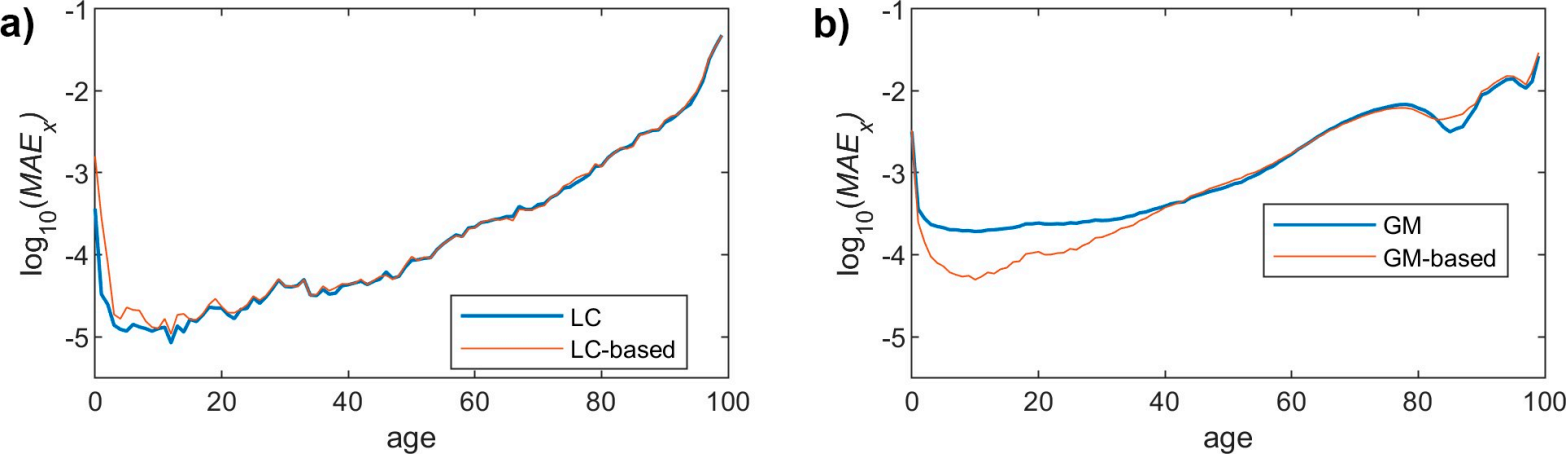
MAE (females) vs. age. Polynomial order = 9. (a) LC and LC-based models. (b) GM and GM-based models.

As was discussed before, we focus on the results obtained for the female population, since we have already assessed that the performance of the proposed method yields similar results for both men and women. Figs 7, 8 and 9 show the *MAE*_*t*_ for the two models and the different fitting choices, considering women mortality rate. Although both LC and GM yield rather similar results, we can see that the former leads to a slightly better accuracy. As was the case for the *MAE*_*x*_, the impact of the polynomial degree is much more noticeable in the LC model, since the *MAE*_*t*_ clearly decreases when having higher degrees. In the case of GM, we can see that the proposed model gets closer to the original one, when we increase the degree, but the impact is much less relevant. An interesting observation is that, in this particular case (GM), the results yielded by the proposed model are even more accurate than those obtained when using the original approach.

## Global MAE

To have an overall assessment of the performance of the proposed methodology we now introduce the global MAE. We define the MAE as the average absolute error, considering all mortality rates for all years and ages, as follows:

$$MAE=\frac{1}{X\cdot T}\overset{t_{T}}{\underset{t=t_{1}}{\sum }}\overset{x=x_{X}}{\underset{x=x_{1}}{\sum }}\left|{\hat{m}}_{x,t}-m_{x,t}\right|$$

where $\hat{m}\left(x,t\right)$ is the mortality obtained with the proposed model, *m(x*,*t)* is the real mortality rate (given by the Eurostat information), *T* is the number of considered years, and *X* is the number of considered ages.

**Fig 10:**
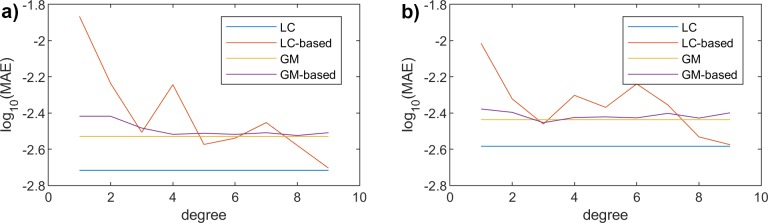
Global MAE vs. Polynomial degree. (a) Females. (b) Males.

Fig 10 shows the global MAE for both traditional models (LC and GM), as well as for the proposed approximations, varying the polynomial degree, for both genders, females and males. We can first observe that the performance of the analyzed models is quite similar for both genders. As we already saw in the previous results, the accuracy of LC is slightly higher, being the global MAE lower: 0.0019 for LC, and 0.0029 for GM. On the other hand, as was already seen for both MAE_X_ and MAE_T_, the impact of increasing the degree of the fitting functions is much more noticeable for the LC model. The global MAE clearly decreases for the LC when the degree is higher, for both genders. However, when using GM, the accuracy of the proposed methodology does not depend too much on the polynomial degree, being reasonably good, even for degree = 1.

Obviously, there exists a trade-off between the accuracy of all models, and their complexity. We assess this latter parameter according to the information that is required. Table 1 shows the global MAE for all models and for three different polynomial degrees (1, 3 and 9) used in the proposed methodology. As was seen before, the original LC model yields the highest accuracy, requiring 236 coefficients, which correspond to *a* (100 values), *b* (100 values) and *k* (36 values). On the other hand, the traditional GM model, exhibiting a reasonably good precision, needs fewer coefficients (108), corresponding to its three parameters *α*, *β* and *γ*, for each year (36 in our case). The complexity of the different approximations depends on the number of coefficients. As a general rule, we can conclude that if we would need to strengthen the accuracy of the model, we should probably use LC and a higher degree. On the other hand, if the requirements on precision were a bit looser, and we wanted to reduce the complexity, we would be able to use GM, even with a low degree.

**Table 1:** Global MAE and complexity of the models.

	Females		Males	
	Global MAE	Number of coefficients	Global MAE	Number of coefficients
**GM-based (1)**	0.0038226	6	0.004190836	6
**GM-based (3)**	0.003288338	12	0.003522342	12
**GM-based (9)**	0.003100473	30	0.003992672	30
**GM**	0.002956596	108	0.003657113	108
**LC-based (1)**	0.013601402	6	0.009657776	6
**LC-based (3)**	0.003116171	12	0.003462138	12
**LC-based (9)**	0.001976763	30	0.00266077	30
**LC**	0.001920282	236	0.002608832	236
**Eurostat data**	0	3600	0	3600

## Conclusions

We have proposed and validated an alternative methodology to establish the mortality rate for a certain geographical area. Although the methodology is applicable to any model, we start by exploiting two well-known methods, Gompertz-Makeham and Lee-Carter, but opposed to previous works, our main contribution is to pay attention to the evolution of those models’ parameters along the years or ages. As an illustrative example, we have focused in mortality rates in Spain, using real Eurostat data, which covers more than 35 years.

We have used polynomial functions to fit such evolutions, and we have studied the impact of using larger degrees. The results show that the accuracy of the proposed schemes is alike the one that would have been obtained with the legacy approaches, thus validating our proposed methodology.

Our solution has two main advantages compared to traditional solutions: 1) it strongly reduces the number of data that is required to use the model, even when using large degree polynomial fitting functions. 2) On the other hand, since our methodology intrinsically captures the temporary evolution of the mortality rate, it might be used to predict values even for years in which we do not have real data.

Our future work comprises two main lines of research. First, we will exploit the proposed methodology to predict the mortality rate, without knowing real data, which would actually prevent using the legacy approaches. We will study the impact of the configuration of the proposed scheme, by increasing the fitting function degree, and changing the number of years used to obtain such fitting function. We will as well assess the performance of the proposed methodology for other geographical areas. On the other hand, our proposal is generic enough to apply it to other social parameters, like the fertility rate. In this sense, our ultimate goal is to develop a tool to generate realistic social networks interactions, and a first step towards such objective is to start by creating artificial populations, which evolve (fertility and mortality rates) similarly to real ones. We will thus exploit the proposed methodology as a module within such framework.
